# A recurrent GNE variant causing GNE myopathy in unrelated patients from Pakistan: a case series

**DOI:** 10.1186/s13256-025-05524-4

**Published:** 2025-08-30

**Authors:** Shafaq Saleem, Fizza Akbar, Salman Kirmani, Ehtesham Khalid, Sara Khan

**Affiliations:** 1 Neurology Department, Aga Khan University and Hospital, Main Campus, Karachi, Pakistan; 2 Division of Women and Health, Aga Khan University and Hospital, Main Campus, Karachi, Pakistan; 3 Ochsner Health, New Orleans, LA USA

**Keywords:** GNE myopathy, P. Val727Met, Distal myopathy, Founder variant, Next generation sequencing, Neuromuscular disease

## Abstract

**Background:**

GNE myopathy, also referred to as bifunctional UDP-N-acetylglucosamine 2-epimerase/N-acetylmannosamine kinase myopathy, is a progressive distal myopathy marked by rimmed vacuoles and linked to a variety of disease-causing genetic variants. These variants display considerable ethnic diversity worldwide. However, no studies to date have explored GNE disease variants in the Pakistani population.

**Case presentation:**

We report four unrelated adolescent male patients, from Pakistan, diagnosed with GNE myopathy. All four patients shared variant, c.2179G > A (p. Val727Met), which has previously been observed in Indian populations. Among these patients, three exhibited the variant in a compound heterozygous state along with a second variant; three out of four patients were born to consanguineous parents with positive family history of similar weakness in all cases described. Symptoms in these patients began at an average age of 21.5 years; three of the four patients became wheelchair dependent within 10 years from symptoms onset.

**Conclusion:**

The GNE variant c.2179G > A (p. Val727Met) is not exclusive to the Indian Rajasthani population but could also be prevalent in Pakistan, likely owing to shared South Asian ancestry. This report represents the first case series from Pakistan focusing on a specific GNE variant, providing a valuable addition to the genetic understanding of GNE myopathy in this population. This finding underscores the need for further genetic studies to explore the presence and impact of GNE variants in Pakistan and neighboring South Asian regions.

## Background

*GNE* (bifunctional UDP-N-acetylglucosamine 2-epimerase/N-acetylmannosamine kinase) myopathy or distal myopathy with rimmed vacuoles, is a rare autosomal recessive progressive distal myopathy, associated with a wide range of disease-causing variants in *GNE*. This adult-onset disorder has over 200 identified disease-causing variants and the number is likely to expand with advent of improved molecular diagnostics through Next Generation Sequencing (NGS) [1]. Disease-causing variants showing ethnic diversity are found in different parts of the world [2]. Despite multiple genetic variants, phenotype remains similar with clinical weakness starting in early adulthood, at an average age of 26 years, involving distal muscles and classically sparing the quadriceps [3]. Molecular analysis has identified multiple genetic variants with some variants showing prevalence in select ethnicities and some founder variants well-studied in different populations. Molecular analysis has confirmed that Met743Thr is most prevalent among the non-Jewish Middle Eastern populations. In patients of Japanese ancestry, multiple variants including Cys44Ser, Asp207Val, and Val603Leu, and in the Roma Bulgarian population variant Ile618Thr are commonly observed [4]. Variant p.Val727Met has been reported in different parts of the world, however, it is more commonly observed within the South Asian population. Khadilkar et al. reported Val727Met as the most common variant in the Rajasthan population [1]. A thorough literature review did not reveal any studies examining genetic variants specifically in GNE patients from Pakistan.

In this report, we present four patients (see Table 1) who possess a common variant, c.2179G > A, p. Val727Met, previously recognized as a prevalent GNE variant in the Indian Rajasthani population. **Patients were enrolled in this study after providing written informed consent. Electromyography was performed using the Nicolet Viking (Viasys Healthcare Inc., USA) system with a concentric needle. Muscle evaluation followed our institutional myopathy protocol, which comprehensively examines seven to eight upper and lower limbs muscles.** This case series highlights the genetics of distal hereditary myopathy in Pakistan, demonstrating that the same genetic variant can occur in unrelated families. Moreover, this marks the first documented identification of a potentially common GNE variant within the Pakistani population, indicating a shared genetic element in GNE-related myopathy across these regions. This finding offers valuable insights into the genetic landscape of hereditary myopathy in Pakistan and may pave the way for further research into genetic similarities and differences within South Asian populations.

**Table 1:** Clinical details of patients with GNE mutation-positive

PATIENT ID	1	2	3	4
Symptom onset	25 years	25 years	20 years	16 years
Consanguinity	Yes	Yes	Not Known	Yes
Family history	Yes	Unknown	Yes	Yes
Symmetry/dorsiflexors powers	Symmetrical/foot dorsiflexors (0/5)	Asymmetrical/foot dorsiflexors (2/5)	Symmetrical/foot dorsiflexors (0/5)	Symmetrical/foot dorsiflexors (1/5)
CPK (IU/L)	589	1422	537	208
EMG-NCS	Irritable myopathy affecting both upper and lower limbs	Myopathic potential and no active denervation	Not known	Irritable myopathy
Molecular result	GNE-related myopathy	GNE-related myopathy	GNE-related myopathy	GNE-related myopathy
Variants/zygosity/gene/variant classification	c.922C > T (homozygous/GNE/pathogenic) c.2179G > A (homozygous/GNE/likely pathogenic) c.613C > T (heterozygous/KLHL40/uncertain significance)	c.2179G > A (heterozygous/GNE/likely pathogenic) c.559 T > C (heterozygous/GNE/uncertain significance) c.5771A > G (assumed compound heterozygous/NEB/variant of uncertain significance)	c.2179G > A (heterozygous/GNE/likely pathogenic) c.1A > G (heterozygous/GNE/variant of uncertain significance)	c.650A > G (heterozygous/GNE/pathogenic) c.2179G > A (heterozygous/GNE/likely pathogenic) (confirmed compound heterozygous)

## Case presentation

Case 1: a male patient in his 20s of Pakistani descent presented with recurrent falls owing to foot drop, which began in early adulthood. Clinical examination revealed complete paralysis of bilateral foot dorsiflexion with a strength of 0/5 on the MRC scale, while plantar flexion was rated 2/5. Proximal muscle involvement was noted, with hip flexors scoring 1/5 and hip extensors 0/5, whereas the quadriceps were relatively spared with a strength of 4/5. Examination of the upper limbs showed muscle strength of 4/5 in all groups, except for both biceps at 3/5 and the right first dorsal interosseous at 0/5. No weakness was observed in the neck flexors, cranial nerves, or bulbar muscles, and sensory examination revealed no abnormalities.

His condition progressively worsened, and he became wheelchair-bound 12 years after the onset of symptoms. He was born to consanguineous parents, and both his brother and maternal uncle displayed similar symptoms. His creatinine kinase (CK) levels were measured at 589 I.U./L. Electromyography (EMG) indicated signs of irritable myopathy (short MUAP, early recruitment and denervation changes), though muscle biopsy and MRI muscle were not performed. Genetic testing identified two distinct homozygous missense pathogenic (P) and likely pathogenic (LP) variants in the GNE gene: c.922C > T, resulting in the protein change p. Arg308Cys, and c.2179G > A, leading to p. Val727Met consistent with the diagnosis of GNE myopathy. The third variant, c.613C >, was acquired in heterozygous state with variant classification of uncertain significance, not sufficient enough to determine the role of this variant in the disease.

Case 2: a male in his early 20 s, of Pakistani origin, presented with progressively increasing recurrent falls with imbalance while walking and climbing stairs. Clinical examination demonstrated intact upper limb muscle strength, with all groups scoring 5/5 on the MRC scale. In the lower limbs, there was asymmetrical weakness, more pronounced on the left, with dorsiflexion strength rated 2/5 on the left and 1/5 on the right, while hip flexion was 3/5. The remaining lower limb muscle groups, cranial nerves, bulbar muscles, and neck flexors were preserved. Sensory examination revealed no deficits.

The weakness progressed to wheelchair dependency within 8 years. Family history was positive in siblings with a similar illness. CK was 537 I.U./L. Electromyography showed myopathic features (short MUAPs and early recruitment pattern) with no active denervation changes. Genetic testing showed two heterozygous variants c.2179G > A (p. Val727Met) and c.559 T > C (p. Tyr187His) in *GNE*, both variants were also identified in the symptomatic sibling of the proband, assumed to be present in a compound heterozygous state though parental testing was not done to confirm the zygosity. Variant c.559 T > C (p. Tyr187His) and c.5771A > G (p. Tyr1924Cys were classified as a variant of undetermined significance (VUS); however, clinically it was very suspicious to be leading to the patient phenotype, thus likely classified as uncertain significance.

Case 3: a male in his early 20 s, of Pakistani descent, presented with difficulty walking and recurrent falls. His symptoms began during adolescence and progressively worsened. Clinical examination revealed powers of 3/5 in bilateral biceps muscle and intact powers in the remaining upper limb muscle group according to MRC scale. Lower limb power grading severely affects bilateral ankle dorsiflexion (0/5) with relative less involvement of proximal muscles of lower limbs. Examination showed hip flexors exhibiting (2/5), hip extension (4/5) and quadriceps and planter flexion (5/5). No cranial, bulbar, or sensory deficit was revealed.

He became wheelchair-bound within 10 years of symptom onset. He was born to consanguineous parents, and his maternal uncle was similarly affected. His creatine kinase level was 1422 I.U./L, and no electromyography records were available. Genetic testing identified two heterozygous variants: c.2179G > A (p. Val727Met) and c.1A > G (p.?), later of which was classified as a variant of uncertain significance (VUS) but suspected to be disease-causing due to its effect on the start codon of the gene. c.2179G > A (p. Val727Met) was acquired in heterozygous fashion classified as the disease-causing variant. While parental testing could not confirm the zygosity of the variants, they were assumed to be in a compound heterozygous state.

Case 4: a male in his early 20 s, of Pakistani descent, presented with difficulty running and climbing stairs. His symptoms began in adolescence and have progressively worsened over the years. Clinical examination revealed severe, symmetric weakness in bilateral ankle dorsiflexion, with muscle strength rated 1/5 on the MRC scale, while proximal muscle strength was relatively preserved at 4/5. There was no involvement of the upper limb musculature, neck flexors, bulbar muscles, or cranial nerves. Creatine kinase levels were measured at 208 I.U./L. He was born to consanguineous parents, and his paternal uncle has a similar history of weakness.

His current dependency status is unknown. Electromyography indicated signs of an irritable myopathy (short MUAPs, early recruitment and denervation changes). Genetic testing revealed compound heterozygous pathogenic variants: c.2179G > A (p. Val727Met) and c.650A > G (p. Tyr217Cys). The former was identified in a pathogenic state, aligning with the patient’s clinical symptoms, while the latter was classified as a likely pathogenic variant. Since the family was not screened, determining the pathogenicity of c.650A > G through clinical correlation remained challenging.

## Discussion

GNE myopathy, also known as hereditary inclusion body myopathy, is a rare autosomal recessive disorder characterized by progressive distal muscle weakness and often begins in early adulthood. The disease has an estimated prevalence of 1 in 9 million [4], with pathophysiology hypothesized to involve hypo-sialylation of muscle glycans necessary for sialic acid synthesis. This results in the accumulation of abnormal protein aggregates within the muscle cells, contributing to muscle fiber damage and degeneration. To date, over 200 pathogenic variants have been identified in the GNE gene worldwide, with specific variants showing notable ethnic and regional variability.

Zhao J et al. reported 16 novel pathogenic or likely pathogenic (P/LP) variants in unrelated individuals with GNE myopathy, indicating the genetic diversity of this disorder. In the Chinese population, common variants include p. Asp207Val (p.D176V) [5] and p.D207V [6–8], both associated with a milder phenotype and a later age of onset compared with other variants. In Japan, p. V603L, p. Asp409Tyr, and p.Ala662Val are frequently observed, with p.V603L noted as the second most common variant [9]. This Japanese variant pattern is associated with more severe symptoms and rapid disease progression, making it distinct from the presentation seen in other populations. Similarly, in the Turkish population, a unique homozygous P/LP variant (c.2152G > A, p.A718T) was identified in two sisters, presenting with classical GNE myopathy features on muscle biopsy [2]. In Bulgaria, the p.I618T variant is a founder mutation seen across all known GNE myopathy cases [10]. Meanwhile, p.M743T is frequently observed in patients from Kuwait [11] and two unrelated Tunisian families [12]. Additionally, Chaouch et al. identified p. Ala662Val and p. Asp409Tyr as prevalent GNE variants in Northern Irish and North English populations [13]. In India, Nalini et al. identified p. Val727Met as a common variant, and this variant has also been observed in Thai individuals [14, 15], suggesting that it may be widespread among South Asian populations. **Similarly, the Middle Eastern population exhibited a high prevalence of homozygosity for the M712T mutation, with varying symptoms among individuals **[16].

The relationship between genotype and phenotype in GNE myopathy remains challenging to define, mainly due to underdiagnosis, limited genetic testing, and a lack of comprehensive studies on the disease’s natural history. Some studies suggest that certain variants, like D207, are associated with a later onset of symptoms [14], whereas others, like c.830G > A (p.R277Q), show a tendency toward earlier and more severe disease manifestation. This complexity is evident in our patient series, where those carrying the p. Val727Met variant exhibited an average age of onset of 21.5 years and displayed a moderate to slow disease progression. Among these patients, two became wheelchair-dependent within approximately 8 years of symptom onset, with all four experiencing predominantly distal muscle weakness. Electromyography and nerve conduction studies (EMG/NCS) confirmed classic myopathic changes in three patients with concomitant active denervation changes in two patients, corroborating existing literature on variation in hereditary myopathy.

Our findings support existing literature, which documents phenotypic variability in GNE myopathy, even among individuals with identical genetic mutations. Patients harboring the p.V603L variant in a Japanese cohort exhibited an earlier onset and more severe progression [17], whereas those with p. Val727Met in our series showed slower progression. Some patients with GNE myopathy display typical myopathic findings, such as fibrillation potentials and positive sharp waves in the anterior lower limb compartment [18], while others may present with atypical neuropathic features [19]. This variability highlights the complex genotype–phenotype relationship in GNE myopathy and underscores the need for more extensive studies to understand its clinical spectrum better*.*

Creatine kinase (CK) levels in myopathies generally indicate the extent of muscle damage and can serve as a marker of disease activity. In the early stages of GNE myopathy, CK levels are typically below 1000 IU/L (4), although they may rise into the thousands as the disease progresses. However, elevated CK levels are not consistently observed in all cases. Studies have documented normal CK levels even in patients showing clinical signs consistent with GNE myopathy (13). While CK measurements can reflect muscle damage, variability in CK levels does not necessarily correlate with disease severity or progression**.**

Our cases involve four unrelated individuals from Pakistan with GNE myopathy who carry the disease-causing variant p. Val727Met. This variant has been identified as a founder variant in the Rajasthani population and is recognized as the most common GNE myopathy variant in India [1]. Notably, this same variant was also identified in two Asian-born patients in a 2014 study conducted on the British population. These findings suggest that the c.2179G > A, p. Val727Met variant is likely a founder mutation within the South Asian population, contributing to GNE myopathy in this demographic.

## Conclusion

*GNE* c.2179G > A, p. Val727Met is not just specified to the Rajasthani population but also shows a possibility of being a common variant even in the Pakistani population. As the population of the subcontinent from India and Pakistan shares a common ancestry, it is plausible that other common founder variants in genetically heterogeneous neuromuscular disorders are present and remain to be explored. As the cost of genetic testing continues to decrease and molecular analysis becomes an integral part of a patient’s diagnostic pathway, our understanding of ethno-specific disease-causing variants and their genotype–phenotype correlation will continue to expand; subsequently leading to improved patient care.

## Data Availability

Data will be made available on reasonable request by the corresponding author.

## Abbreviations

GNE: UDP-N-acetylglucosamine 2-epimerase/N-acetylmannosamine kinase
NGS: Next Generation Sequencing
CK: Creatinine Kinase
MRI: Magnetic Resonance Imaging
EMG: Electromyography
NCS: Nerve Conduction Studies
VUS: Variant of Undetermined Significance
MRC Scale: Medical Research Council Scale
